# Interaction of Cowpea Mosaic Virus (CPMV) Nanoparticles with Antigen Presenting Cells In Vitro and In Vivo

**DOI:** 10.1371/journal.pone.0007981

**Published:** 2009-11-23

**Authors:** Maria J. Gonzalez, Emily M. Plummer, Chris S. Rae, Marianne Manchester

**Affiliations:** Department of Cell Biology, The Scripps Research Institute, La Jolla, California, United States of America; Federal University of São Paulo, Brazil

## Abstract

**Background:**

Plant viruses such as *Cowpea mosaic virus* (CPMV) are increasingly being developed for applications in nanobiotechnology including vaccine development because of their potential for producing large quantities of antigenic material in plant hosts. In order to improve efficacy of viral nanoparticles in these types of roles, an investigation of the individual cell types that interact with the particles is critical. In particular, it is important to understand the interactions of a potential vaccine with antigen presenting cells (APCs) of the immune system. CPMV was previously shown to interact with vimentin displayed on cell surfaces to mediate cell entry, but the expression of surface vimentin on APCs has not been characterized.

**Methodology:**

The binding and internalization of CPMV by several populations of APCs was investigated both *in vitro* and *in vivo* by flow cytometry and fluorescence confocal microscopy. The association of the particles with mouse gastrointestinal epithelium and Peyer's patches was also examined by confocal microscopy. The expression of surface vimentin on APCs was also measured.

**Conclusions:**

We found that CPMV is bound and internalized by subsets of several populations of APCs both *in vitro* and *in vivo* following intravenous, intraperitoneal, and oral administration, and also by cells isolated from the Peyer's patch following gastrointestinal delivery. Surface vimentin was also expressed on APC populations that could internalize CPMV. These experiments demonstrate that APCs capture CPMV particles *in vivo*, and that further tuning the interaction with surface vimentin may facilitate increased uptake by APCs and priming of antibody responses. These studies also indicate that CPMV particles likely access the systemic circulation following oral delivery via the Peyer's patch.

## Introduction

Viruses are increasingly being used in nanotechnology applications for a variety of purposes as diverse as material science, vaccine development, and therapeutic design. For many years animal viruses have been developed for gene delivery and gene therapy purposes [1]. More recently, other pathogens such as plant viruses, bacteriophages and viruses of Archaea are increasingly being used for nanobiotechnology purposes because of their relative structural and chemical stability, ease of production, and lack of toxicity and pathogenicity in animals or humans [2], [3], [4], [5]. In particular, plant viruses have been at the forefront of efforts to develop novel vaccines, in part because of the potential for producing large quantities of antigenic material in plant hosts, as well as the possibility for developing orally-bioavailable antigens. A variety of plant pathogens such as cowpea mosaic virus (CPMV) [2], [3], [4], [5], [6], [7], cucumber mosaic virus (CMV) [8], [9], alfalfa mosaic virus (AlMV) [10], tobacco mosaic virus (TMV) [11], [12], [13] and papaya mosaic virus (PapMV) [14] have been exploited for vaccine purposes, whereby the viruses are modified to present antigens through genetic introduction of foreign epitopes and proteins, thus combining engineering of the multivalent antigen with large-scale production of the antigen in plants.

CPMV-based vaccines in particular have been shown to induce neutralizing antibodies against multivalently-displayed epitopes derived from infectious agents [15], [16], [17], [18]. Advantages of using CPMV particles as a vaccine delivery system include the natural resistance of CPMV to high temperature, low pH and proteolysis as well as the potential for large-scale production in the natural host black-eyed pea or *Vigna unguiculata* (≥1.0 g of virus per kilogram of leaves). CPMV, a comovirus that encodes a 31 nm capsid, is a member of the picornavirus superfamily of viruses. CPMV capsids are composed of 60 copies of the large (L) and small (S) protein subunits assembled around an RNA genome [16], [18], [19]. Antigenic epitopes are commonly displayed by introduction of peptide sequences in regions of the genome encoding the capsid surface loops. In addition, the surface may be chemically modified for direct attachment of peptides and other ligands [20], [21], [22], [23]. A variety of bioconjugation methods have been developed, with surface lysines or introduced cysteines most typically utilized.

CPMV chimeric viruses displaying foreign epitopes from pathogens including HIV-1 [6], [7], mink enteritis virus [17], *Staphylococcus aureus* [4], [5] and *Pseudomonas aeruginosa* [3], [5] induce strong humoral immune responses against those pathogens *in vivo*, some of which induce protective levels of immunity. It is thought that the multivalent nature of antigen display on the virus particle surface facilitates efficient induction of epitope-specific responses. Nevertheless, relatively little is known about how CPMV or other plant viruses interact with antigen presenting cells (APCs) either *in vitro* or following delivery *in vivo*.

Previously, we studied the bioavailability of CPMV *in vivo* after oral or intravenous delivery in mice. CPMV has been extensively studied *in vivo* [24], [25]. We demonstrated that following delivery in mice, CPMV was found in virtually all tissues for several days after administration [26]. In addition, we showed that CPMV was stable under gastric conditions, and following oral delivery we also observed virus throughout the mice suggesting that the virus had crossed the gastrointestinal epithelium, perhaps by interaction with Peyer's patches (PP). These secondary lymphoid organs are located in the terminal ileum of the small intestine and contain dendritic and other cells of the immune system. CPMV was also detected in spleen and lymph nodes, however the individual cell types that interacted with virus were not determined [26].

The potential for the use of CPMV and other plant virus particles for *in vivo* vaccine and other applications require a better understanding of interactions with APCs *in vitro* and *in vivo*. The most efficient, or “professional” APCs include dendritic cells (DCs), macrophages and B cells. These cell types are optimally equipped to present antigens to T-cells primarily because of their capabilities in antigen capturing. DCs are the most potent APCs because they are highly specialized in internalizing and processing antigens. Immature DCs (imDCs) ingest antigens via a number of mechanisms, including receptor-independent macropinocytosis, receptor-dependent phagocytosis, and receptor-mediated endocytosis via clathrin-coated pits or caveolae [27], [28], [29], [30], [31], [32]. Mature DCs (mDC) express high levels of co-stimulatory molecules CD40, CD80 and CD86, which synergize with antigen in the activation of naïve T-cells. In the mouse there are three known DC subsets in the spleen and three in the intestinal PP, and these are discernable based on surface marker expression. CD8α^+^ and CD8α^−^ DC subsets are often considered to be of lymphoid and myeloid origin respectively, although the developmental origin of these cells remains controversial [33], [34], [35]. The mouse spleen contains myeloid (CD11c^+^, CD11b^+^, CD8^−^) lymphoid (CD11c^+^, CD11b^−^, CD8α^+^) and plasmacytoid DC (CD11c^+^, B220^+^), the latter subtype expressing the CD45 isoform (B220) that is normally expressed by B cells [36], [37], [38]. In the intestinal PP, a third subset of CD11c^+^ DCs distinct from lymphoid and myeloid phenotypes, has been characterized which does not express either high levels of CD11b or CD8α molecules and are thus defined as double negative DCs (CD11b^−^, CD8α^−^) [39], [40]. Macrophages, natural killer (NK) cells, and B-cells also can act as APCs, albeit with less potency than DCs [41], [42].

The ability of CPMV to bind and be internalized by several populations of APCs was characterized *in vitro*. The uptake of CPMV particles by APCs *in vivo* following intravenous, intraperitoneal or oral dosing was investigated by flow cytometry and fluorescence confocal microscopy. Next, the association of CPMV particles with gastrointestinal epithelium and PPs was examined by ileal loop ligation and confocal microscopy. Finally the expression of CPMV binding proteins on APCs was evaluated by flow cytometry.

## Results

### APCs Bind and Internalize CPMV Particles In Vitro

Several fluorescently-labeled versions of CPMV were made to visualize CPMV by fluorescence microscopy and detect it by FACS. Fig. S1A shows a space-filling model of CPMV with the 300 naturally-occurring exposed lysine residues labeled in red, although typical labeling using NHS-ester forms of fluorescent dyes yields a maximum of 240 dyes/particle [20]. For most of the experiments Alexa Fluor 488 (AF488) was conjugated to the particles, which has a stronger and more stable signal at varying pH. The lysine residues were conjugated with NHS-AF488, obtaining an average of 71.33 dyes per particle to generate CPMV-AF488. For some studies a cysteine mutant labeled with maleimide-fluorescein was used as well. Fig. S1B shows a genetically modified CPMV (vEFα) containing a cysteine residue in each of the large subunits of the capsid [20], and the 60 addressable cysteine residues are indicated in blue. CPMV-vEFα particles bioconjugated with maleimide-F were labeled at 23 dyes per virus particle to yield CPMV-F. The use of either CPMV-AF488 or CPMV-F in the experiments is noted.

To analyze the ability of APCs to capture and internalize CPMV particles, we used the fibroblast cell lines BalbCl7 and MC57, as well as immature dendritic cells (imDCs) derived from mouse bone marrow, and primary mouse splenocytes. Although the BalbCl7 and MC57 cell lines are not considered professional APCs, they have been used extensively for antigen presentation in cytotoxic T-cell recognition and killing assays [43], [44], [45].

**Figure 1:**
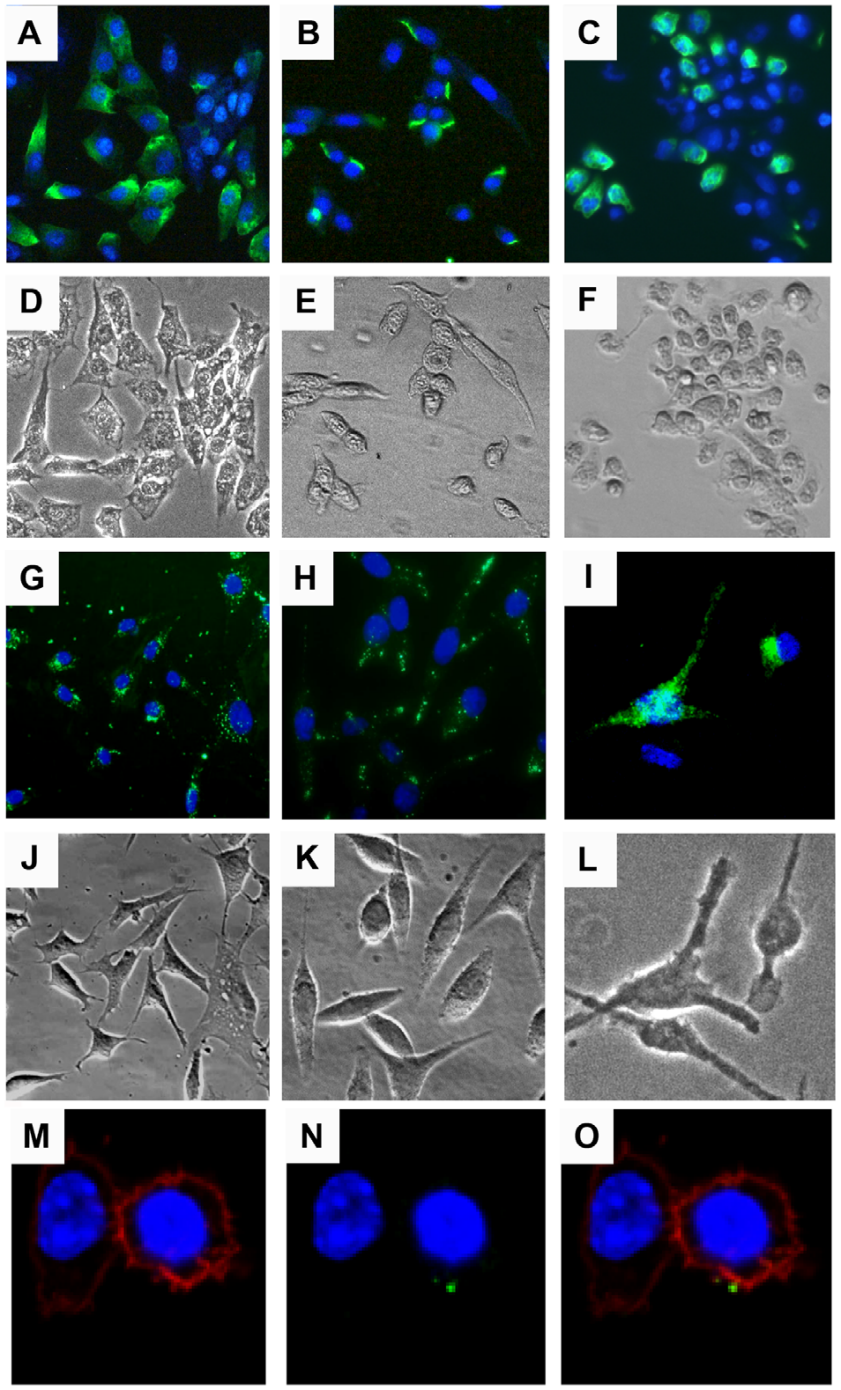
Binding and internalization of CPMV nanoparticles *in vitro*. (A–F) Binding of CPMV to (A) BalbCl7 cells, (B) MC57 Cells, and (C) bone marrow-derived immature DCs (imDC). Cells were fixed with 2% formaldehyde and incubated with CPMV-AF488 (green) on ice overnight. The cells were washed and stained with Hoechst 33258 to visualize the nucleus (blue). (G and H) Uptake of CPMV-F by the cell lines BalbCl7 and MC57, respectively. (I) Uptake of CPMV-AF488 by imDCs. Cells were incubated overnight with the fluorescent virus particles (in green), washed with PBS, fixed, and stained with DAPI (G and H) or Hoechst 33258 (I) to visualize the nucleus (blue). (D–F and J–L) Transmission light image showing the body of the cells. The cells were visualized by fluorescence microscopy using the 20X objective. (M–O) Surface vimentin expression in Caco-2 cells stained with DAPI (blue, M–O), wheat germ agglutinin- AF555 (red, M, O), and specific antibodies to vimentin conjugated to AF647 (green, N–O). Cells were fixed and incubated with WGA one hour RT, anti-vimentin V9 antibody, 1.5 hour RT, and secondary AF647 one hour RT (M–O). Cells were visualized by fluorescence microscopy using the 60X objective (M–O).

CPMV-AF488 was used to study the binding and internalization of CPMV in fibroblast BalbCl7 and MC57 cell lines, and immature murine bone-marrow derived DCs (imDCs). Cells were fixed and incubated with virus particles overnight at 4°C and visualized by fluorescence microscopy. As shown in Fig. 1 (A–F) these three cell types appeared fluorescent on their surface, suggesting that the CPMV particles were able to bind to their membranes. After an overnight incubation the majority of BalbCl7 and MC57 cells were CPMV-positive, but only 40% of the imDC population bound CPMV (Fig. 1A–C). To evaluate the ability of APCs to internalize CPMV particles *in vitro* by fluorescence confocal microscopy, CPMV-F was used to detect the uptake of the particles by BalbCl7 and MC57 cell lines. The cells were cultured overnight with CPMV-F, fixed and stained with DAPI to visualize the nucleus. As shown in Figure 1 (G–H and J–K) both cell lines contained green fluorescent vesicle-shaped structures in the cytoplasm. Bone marrow derived imDCs were also analyzed with the same procedure but using CPMV-AF488. As shown in Figure 1 (I and L) imDCs also internalized CPMV. These results support our previous finding of CPMV particle localization within LAMP-2 and ß-COP-bearing Golgi and lysosome organelles both in BalbCl7 cells [24] and murine DCs [25].

Analysis by flow cytometry and confocal microscopy do not allow confirmation that the fluorescence within the cell is from intact CPMV particles. Endothelial cells and macrophages (antigen-presenting cells which are known to be phagocytic) have been shown by transmission electron microscopy to take up intact CPMV particles [25]. It is possible that, particularly in cell types like NK cells which are not known to have significant phagocytic properties, the fluorescence observed represents virus particle cleavage products or subunits. In any case, it is currently unknown what mechanism antigen-presenting and other cells utilize to internalize CPMV, so the importance of phagocytic ability in this process is not known.

### Quantitation of CPMV Binding and Internalization by DCs In Vitro

Since DCs are the most potent APCs, their capacity to bind and internalize CPMV particles in relation to their maturation state was analyzed further. ImDCs were generated from bone marrow progenitors using granulocyte macrophage colony-stimulating factor (GM-CSF) [46]. After 9 or 10 days of culture 87.7% of the cell population differentiated into CD11c^+^/CD11b^+^ double positive DCs. These cells expressed low amounts of the co-stimulatory molecules CD80 (88.5%) and CD86 (84.1%), and were negative for CD40.

**Figure 2:**
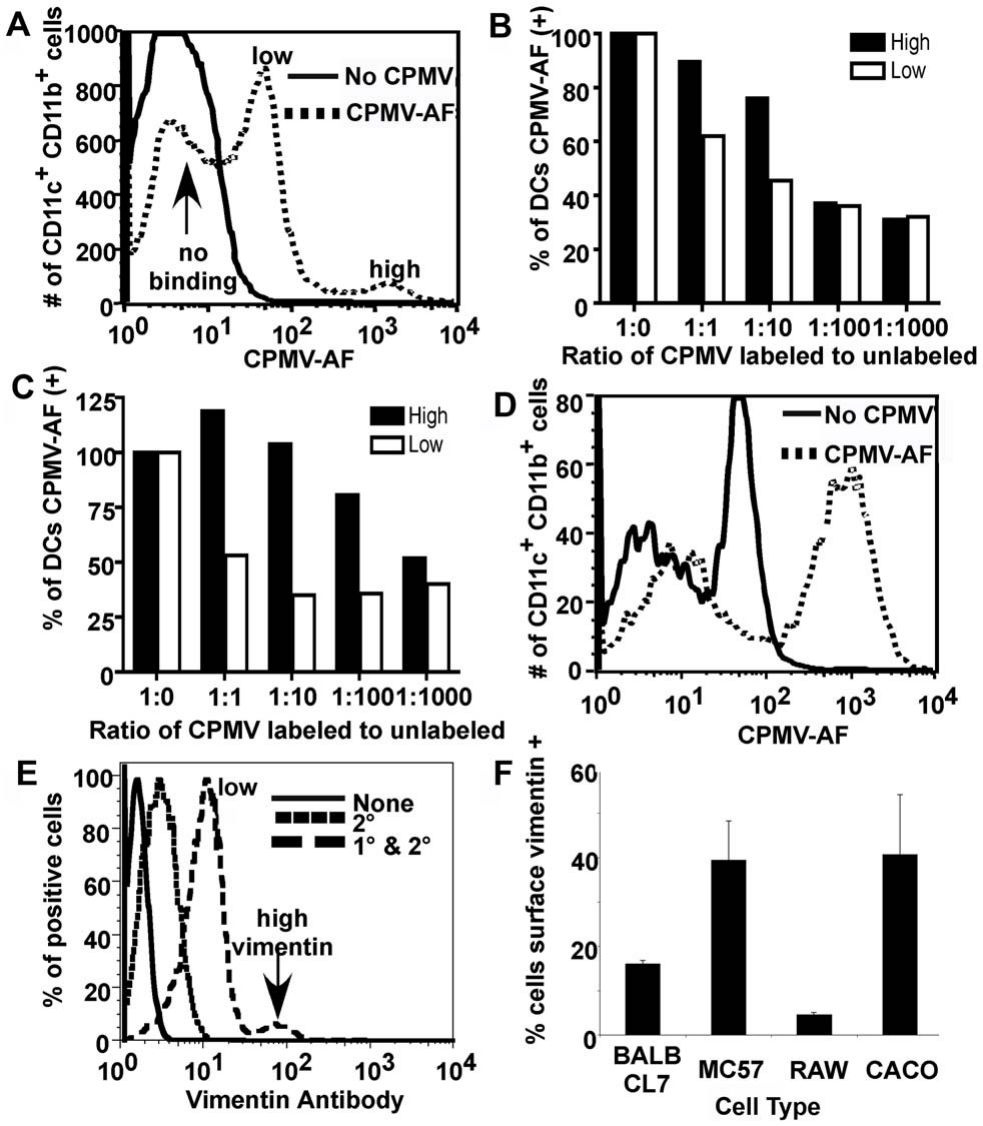
Binding and uptake of CPMV particles by imDCs. (A, B and C) CPMV binding. (A) FACS analysis of a culture of ten days differentiated bone marrow cells. Cells were incubated on ice for 3 hours with CPMV-AF488 (dashed line), washed and stained with APC-CD11c and PE-CD11b antibody markers to identify imDCs. PBS was added to the control cells (solid line). (B and C) CPMV competition assay. After labeling with APC-CD11c and PE-CD11b markers 1×10^6^ cells (B) or 5×10^5^ cells (C) per well were incubated on ice with different amounts of unlabeled CPMV (0 µg, 1 µg, 10 µg, 100 µg and 1000 µg). After washing, 1 µg of CPMV-AF488 was added to each well generating a ratio of 1∶0, 1∶1, 1∶10, 1∶100 and 1∶1000 of CPMV-AF488 to unlabeled CPMV. The black columns represent the high binding imDCs and the white columns represent the low binding imDCs. (D) FACS analysis of CPMV uptake by imDCs. Cells were incubated for two hours with CPMV-AF488 (dashed line) at 37°C and 5% CO_2_, washed and stained with APC-CD11c and PE-CD11b antibodies and analyzed by FACS to quantify the internalization of CPMV particles into CD11c^+^/CD11b^+^ DCs. PBS was added to the control cells (solid line). (E and F) FACS analysis of surface vimentin expression on MC57 (E, F), BalbCL7, RAW macrophages, and Caco cells (F). Cells were incubated one hour, 4°C with anti-vimentin V4630 antibody, then one hour, 4°C with secondary AF647 antibody.

To characterize CPMV binding the cells were incubated with CPMV-AF488 for 3 hours on ice, washed, stained and analyzed by FACS. The results showed that 40% of the imDCs bound the particles, with two populations of cells observed: a small population with high binding (4%; approximately 100-fold higher than background) and a second one with low binding (36%; approximately 10-fold higher than background), (Fig. 2A). Further characterization showed that the majority of cells that bound CPMV expressed higher levels of CD11c (74.0%) and CD11b (84.1%) molecules, while having low expression of CD80 (89.4%) and CD86 (89.7%) (Table 1). The imDC population that did not bind CPMV was 53.7% CD11c^high^, 53.0% CD11b^high^, 89.1% CD80^low^ and 78.3% CD86^low^. These results indicated that the bone marrow derived imDCs that are more likely to bind CPMV have a higher expression of the CD11c and CD11b proteins, but CD11c^high^ and CD11b^high^ cells that do not bind the virus are also present in the culture. These results suggest that not all of the imDCs derived from bone marrow have the ability to bind CPMV possibly because not all of them express the receptor protein vimentin that binds the CPMV particles. Similar results were observed when internalization of the particles was studied (Table 1).

**Table 1:** Percentage of dendritic cell subpopulations positive and negative for CPMV.

	CD11c^high^	CD11c^low^	CD11b^high^	CD11b^low^	CD80^high^	CD80^low^	CD86^high^	CD86^low^
	Binding							
CPMV(+)	74.0	26.0	84.1	15.9	10.6	89.4	10.3	89.3
CPMV(−)	53.7	46.3	53.0	47.0	10.8	89.1	21.7	78.3
	Internalization							
CPMV(+)	88.5	11.5	86.0	13.9	8.4	91.5	5.4	94.5
CPMV(−)	10.6	89.4	1.4	98.5	15.0	85.0	16.3	83.6

FACS analysis of immature dendritic cell subpopulations ability to bind and internalize CPMV-AF488. Subpopulations include high and low expression of CD11c, CD11b, CD80, and CD86. The majority of cells that bound and internalized CPMV expressed higher levels of CD11c and CD11b molecules, while having low expression of CD80 and CD86.

To establish whether the binding of CPMV to DCs is specific, we performed a competition assay using lys-AF488 labeled and unlabeled CPMV particles. DCs were labeled with the cell markers CD11c and CD11b, and 5×10^5^ or 1×10^6^ stained cells were incubated on ice with different concentrations of unlabeled CPMV particles (from 0 to 100 µg of particles, where 1 µg of CPMV contains 1×10^11^ particles). After washing, the cells were incubated with 1 µg of CPMV-AF488 for three hours on ice. The ratios of labeled to unlabeled CPMV particles were 1∶0, 1∶1, 1∶10, 1∶100 and 1∶1000. Due to the presence of two groups of DCs with high and low binding respectively (Fig. 2A), we analyzed these two populations separately. After acquisition, the CD11c/CD11b double positive cells were selected for binding analysis. The results showed a decrease in the binding capacity of the cells to labeled particles as the amount of competing unlabeled particles increased (Fig. 2B and 2C). As expected, DCs showed a greater degree of competition at lower concentrations of unlabeled CPMV particles than did the CPMV high binding DCs. These results confirm that the binding of CPMV to the cells is specific and there is an approximately 1–2 order of magnitude difference in CPMV binding between the two DC populations.

### Vimentin Expression on APCs

In our studies of CPMV trafficking in mouse, we identified a 54 kD cell-surface CPMV binding protein that was expressed on cells with APC activity (MC57) as well as vascular endothelial cells. We have subsequently identified this protein as a surface-expressed form of the cytoskeletal protein vimentin. The binding of CPMV to vimentin was shown to be dose-dependent [47], [48]. Vimentin forms intermediate filaments in the cytoplasm of cells of mesenchymal origin, but a surface-expressed form has recently been demonstrated on cell types including macrophages and T-cells [49], [50]. Presently, it is unknown to what extent APCs in the mouse are able to bind and internalize CPMV and whether this correlates with surface vimentin expression.

Variation in level of binding and internalization of CPMV by different cell types could be explained by variation in the level of surface expressed vimentin. Membrane stain wheat-germ agglutinin (WGA, red) is compared with vimentin staining (green) to verify surface vimentin versus internal vimentin in Caco-2 cells (Fig. 1, M–O).

The distribution of CPMV binding in Fig. 2A, which includes high, low, and no binding populations, could also partially be explained by the partite distribution of surface vimentin expression on cells of the immune system. FACS analysis of MC57 cells reveals that some cells express surface vimentin at high levels, while some express at low levels, and some are indistinguishable from secondary antibody background (Fig. 2E). In fact, about 40% of MC57 and Caco cells express some level of surface vimentin, while an even smaller percentage is shown on BalbCl7 and RAW macrophage cells (Fig. 2F).

### Immature Dendritic Cells and Other APCs Display Surface Vimentin In Vitro

**Figure 3:**
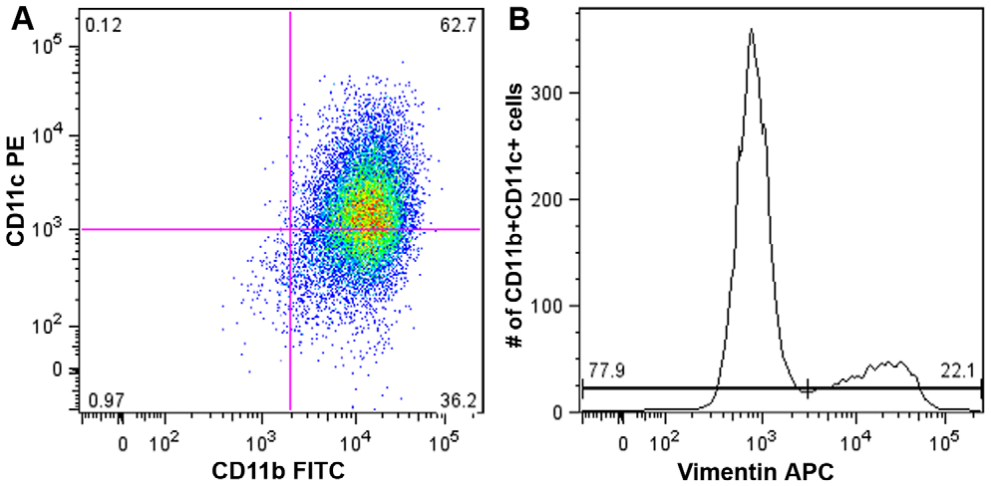
Surface vimentin expression on imDCs. (A) FACS analysis of vimentin on the surface of immature dendritic cells. (B) Surface vimentin positive and negative CD11b^+^ CD11c^+^ cell populations.

After confirming that imDCs were able to bind and internalize CPMV, it became important to investigate whether the CPMV binding protein vimentin was displayed on their surface. ImDCs (62.8% were CD11c^+^/CD11b^+^) were generated from bone marrow by the method described previously. Surface vimentin was stained and the cells were analyzed by FACS. Results indicated that 22.1% of double positive DCs displayed surface vimentin, and the rest were indistinguishable from secondary antibody controls (Fig. 3). These findings confirm that *in vitro*, surface vimentin is present on unactivated DCs, which are able to bind and internalize CPMV. To better define the correlation between vimentin surface expression and CPMV uptake in APCs, BalbCl7 cells were incubated with CPMV for durations of 3 and 24 hours. These cells were then fixed and surface vimentin was stained with specific antibodies. After three hours, 81% of CPMV-AF488 positive cells were also positive for surface vimentin, compared with 51% of the total population of cells (Figure S2). Cells incubated with CPMV-AF488 for 24 hours did not show as strong a correlation (62% of CPMV-AF488 positive cells are positive for vimentin). These results are in good agreement with our previous studies correlating surface vimentin and CPMV binding [47], [48].

### APCs Internalize CPMV Particles In Vivo

**Figure 4:**
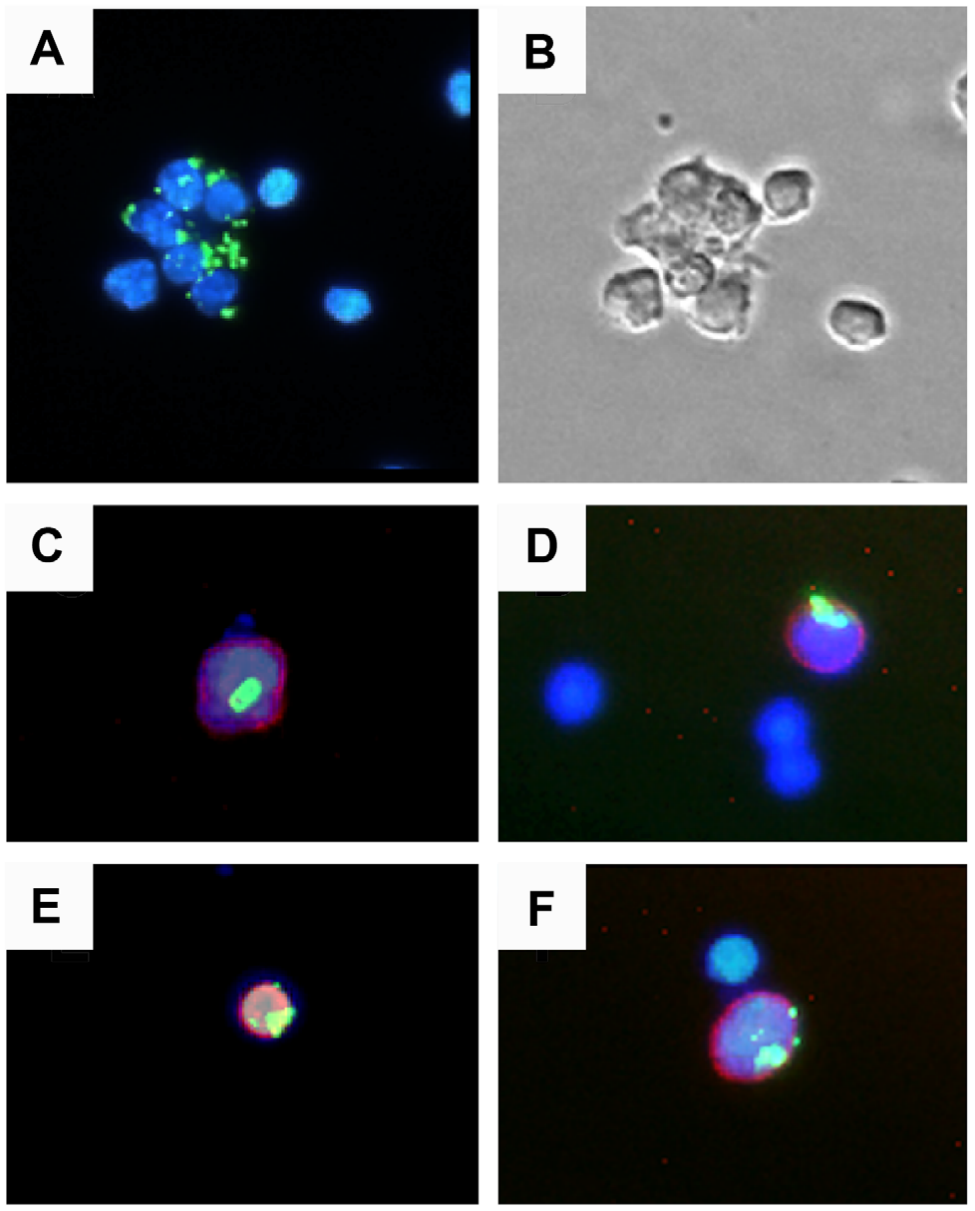
*In vivo* uptake of CPMV particles. (A) Spleen cells were purified 4 hours after i.p. inoculation with 100 µg CPMV-AF488 and stained with DAPI. (B) Transmission light microscopy image showing the body of the spleen cells. (C–F) Localization of CPMV in splenocytes stained with: (C) PE anti-CD8α (red), (D) PE anti-CD11c (red), (E) PE anti-B220 (red), and (F) PE anti NK 1.1 (red). The cell samples were visualized under a fluorescence microscope using a 20X objective. The virus particles are in green and the nuclei are in blue.

To determine whether APCs can internalize CPMV *in vivo*, C57 BL/6 mice were inoculated intraperitonealy using 100 µg of CPMV-AF488 (1×10^13^ virus particles). After four hours, splenocytes were obtained and visualized by fluorescence microscopy. Fig. 4A shows a sample of total splenocytes containing green fluorescence, indicating CPMV uptake. Cell subsets were identified using the antibody markers: PE-CD11c (DCs), PE-CD8α, PE-B220 and PE-NK 1.1 (natural killer cells), and visualized under a fluorescence microscope. As shown in Fig. 4 (C–F), DCs (CD11c^+^), B220^+^ cells, CD8α^+^ cells and natural killer cells (NK 1.1^+^) were able to internalize CPMV *in vivo*.

**Figure 5:**
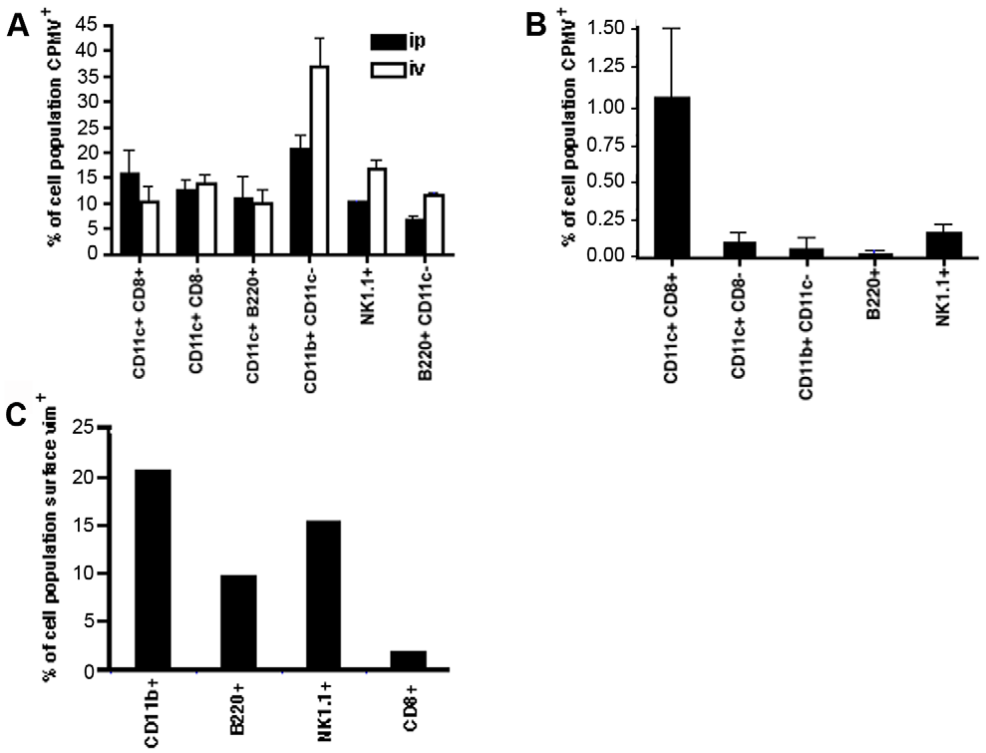
Quantitative analysis of CPMV uptake by different subpopulation of APCs *in vivo*. (A) Groups of three C57BL/6 mice were inoculated: intraperitoneally (black columns) or intraveously (white columns) with 100 µg of CPMV-AF488. After 4 hours, spleens were removed and the cells stained to analyze the internalization of the CPMV into CD11c^+^/CD8α^+^ cells (lymphoid DCs), CD11c^+^/CD8α^−^ cells (myeloid DCs), CD11c^+^/B220^+^ cells (plasmocytoid DCs), CD11c^−^/CD11b^+^ cells (macrophages), B220^+^/CD11c^−^ cells (B-lymphocytes), and NK1.1^+^ cells (natural killer cells). After staining, the cells were fixed and analyzed by FACS. The columns represent the percentage of CPMV positive cells from each subpopulation of cells. Mice inoculated with PBS were used to subtract the background. (B) Groups of three C57BL/6 mice were inoculated by oral gavage using 1000 µg of CPMV-AF488. After 20 hours, spleens were removed and treated the same way as described above. (C) Splenocytes were isolated from C57BL/6 mice and red blood cells were removed. Dendritic cells remain in the connective tissue and do not flow through the cell strainer. Remaining cells were blocked and stained (CD11b for macrophages, B220 for B cells, NK1.1 for natural killer cells, and v4630 anti-vimentin antibody for vimentin). Cells were then fixed and analyzed by FACS.

Based on these findings, the *in vivo* uptake of CPMV particles by different populations of APCs was further quantified after intraperitoneal, intravenous, or oral inoculation. For the first two routes 100 µg of CPMV-AF488 was used and detection was performed after 4 hours, while for the oral route the dose of 1 mg of labeled particles (1×10^14^ virus particles) per mouse was required to detect CPMV in APCs at 20 hrs post-dosing. Splenocytes were obtained, stained and analyzed by FACS to evaluate lymphoid DCs (CD11c^+^ CD8α^+^), myeloid DCs (CD11c^+^ CD8α^−^), plasmasytoid DCs (CD11c^+^ B220^+^), macrophages (CD11c^−^, CD11b^+^), B cells (CD11c^−^ B220^+^), and natural killer cells (NK 1.1^+^). As shown in Fig. 5, all investigated cell populations were able to internalize CPMV particles following each route of administration, corroborating the results observed by immunofluorescence microscopy (as an example, the flow cytometry data for intraperitoneal injection is also shown in Fig. S3). Fig. 5A also shows that the intraperitoneal route generated 5% more CPMV positive lymphoid DCs (CD11c^+^ CD8α^+^) than did the intravenous route, whereas the intravenous route of inoculation resulted in a higher percentage of macrophages, B cells and NK cells internalizing CPMV particles. In comparison with these routes, the amount of cells which had taken up CPMV particles after oral gavage is about 10 fold lower for lymphoid DCs (CD11c^+^ CD8α^+^) and more than 100 times lower for the rest of the cells (Fig. 5B). Moreover, CD11c^+^ CD8α^+^ (lymphoid) DCs take up CPMV relatively more efficiently after oral gavage. The decrease in detection of CPMV positive cells after oral gavage is probably due to partial transport of the particles across the intestinal epithelium or possibly due to partial cleavage of the fluorescent dye from the particles in the gastrointestinal tract. Fig. 5C also confirms that surface vimentin is present on splenocytes including CD8^+^, CD11b^+^, NK1.1^+^, and B220^+^ cell populations.

### CPMV Particles Interact with PP in the Intestinal Epithelium

**Figure 6:**
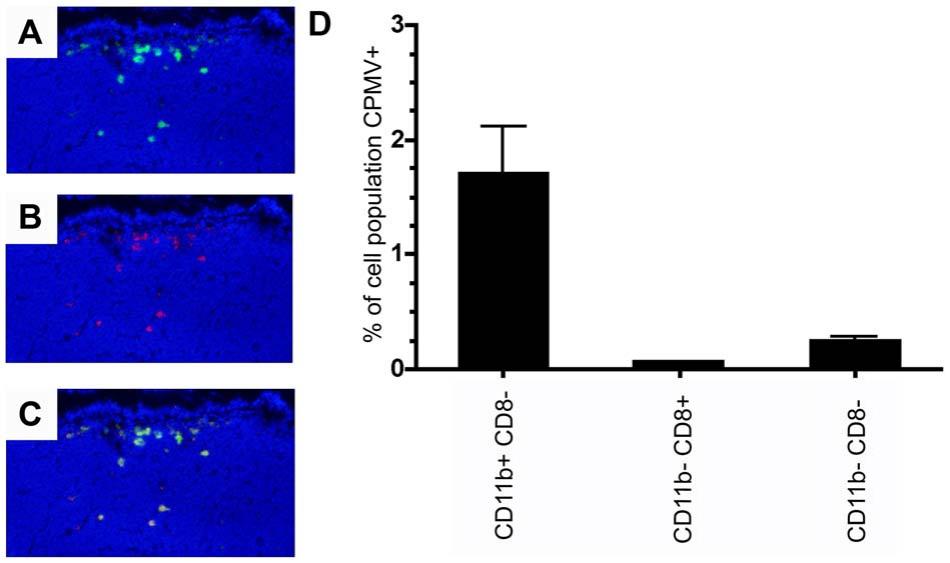
*In vivo* uptake of CPMV particles in intestinal PP. (A) Fluorescence microscopy image showing CPMV-AF488 particles (green) penetrating the dome region of an intestinal PP. (B) Fluorescence microscopy image showing UEA Rhodamine M cell marker (red). (C) Merge A and B. (D) FACS analysis of CD11c positive PP DCs. CPMV positive lymphoid, myeloid and double negative PP DCs expressed as a percent of each respective PP DC subpopulation.

As we had previously shown that CPMV entered the bloodstream following oral inoculation and could be found in splenocytes after oral gavage (Fig. 5), we wished to observe the interaction between CPMV and the intestinal epithelium and Peyer's patch. Ileal loops containing a single PP were injected into the intestinal lumen with 200 µg of CPMV-AF488 and 20 µg UEA rhodamine, a lectin that specifically binds to M cells. As shown in Fig. 6A, CPMV particles were co-localized with the UEA lectin in the sub-epithelial dome (SED) of the PP, indicating that CPMV crossed the intestinal barrier through M cells localized on the follicle-associated epithelium (FAE). To investigate which types of cells within the PP associated with CPMV particles, the PP cells were analyzed by flow cytometry. After incubating the ileal loops with 200 µg of CPMV-AF488 for 1 hour, the cells from two individual PP were stained and analyzed by FACS to evaluate lymphoid DCs (CD11c^+^ CD11b^−^ CD8α^+^), myeloid DCs (CD11c^+^ CD11b^+^ CD8α^−^), double negative DCs (CD11c^+^ CD11b^−^ CD8α^−^) and macrophages (CD11c^−^, CD11b^+^). The results indicate that the myeloid DC subpopulation primarily takes up CPMV in the PP. Macrophages did not take up CPMV particles in the PP (not shown). However, from the total DC population (CD11c^+^ cells) in the PP, the percentage of myeloid and double negative DCs taken up CPMV particles was similar (not shown). The results suggest that the route of particle administration influences the cellular subtypes that are accessible to CPMV. We also demonstrated that Caco-2 cells, a gastrointestinal epithelial cell line similar to M cells, display vimentin on the cell surface and a subpopulation is able to bind and internalize CPMV particles *in vitro* (Fig. 1, M–O). Together these results indicate that a variety of APC populations in the PP and spleen are capable of internalizing CPMV, and the interaction with CPMV is consistent with display of the CPMV-binding protein, surface vimentin.

## Discussion

This study shows that *in vitro*, CPMV binds to and is internalized by APCs such as BalbCl7, MC57 and imDCs derived from mouse bone marrow. Moreover, DCs, macrophages, B cells and NK cells have the ability to capture CPMV particles *in vivo* following intravenous, intraperitoneal, and oral administration. Interaction of CPMV and APCs correlates with surface expression of the CPMV binding protein, surface vimentin. Following oral administration, CPMV associated with the gastrointestinal epithelium, in particular PP. CPMV could also be detected in APCs isolated from PP. Together these results indicate that CPMV can interact with APCs by a variety of routes of administration.

Studies of CPMV binding and internalization by flow cytometry showed that APCs interacted with CPMV. *In vitro*, only about 40% of the imDCs (CD11c^+^ and CD11b^+^) were CPMV positive. The capacity of the imDCs to bind CPMV is not related to the degree of differentiation or level of expression of the CD11c or CD11b markers, since 53% of the imDC population that did not bind CPMV expressed high levels of these molecules. Instead, this capacity is likely better related to the presence and level of expression of the CPMV binding protein, vimentin, on the cell surface. We have shown that *in vitro*, BalbCl7 cells which have recently (3 hr incubation) internalized CPMV particles are more likely to have vimentin on their surface (81%) than those which have have incubated for 24 hours (62%). However, each of the CPMV-AF488 positive populations are more likely to display surface vimentin than the total population of BalbCl7 cells displaying surface vimentin (51%). ImDCs are specialized to take up antigens, and we have demonstrated 88.5% of bone marrow-derived imDCs expressing high levels of CD11c and CD11b molecules efficiently internalized CPMV particles. As we expected, CPMV particles were also efficiently internalized by DCs after *in vivo* inoculation. Following i.p. or i.v. routes of inoculation, CPMV particles were present in lymphoid, myeloid, and plasmocytoid DC populations and after oral gavage the lymphoid cells in the spleen had the highest percentage of internalization.

A variety of virus-like particles (VLPs), and non-viral synthetic nanoparticles have been shown to interact with APCs [14], [51]. Porcine parvovirus (PPV) VLPs are captured by lymphoid and myeloid DCs after iv inoculation [51], papaya mosaic virus (PapMV) VLPs are captured by CD11c^+^ DC after iv inoculation [14] and the polystyrene nanoparticles are taken up into pulmonary DCs following intranasal administration. PPV, PapMV VLPs and polystyrene nanoparticles can, much like CPMV, act as potent vaccines able to generate humoral immune responses [52] and can also induce cellular immunity [14], [51], [53]. Previous studies have also shown the capacity of imDCs to internalize other types of particulate antigens *in vitro*, including gelatin nanoparticles (88%), polystyrene nanospheres and poly (D, L-lactic acid-co-glycolic acid) (PLGA) nanoparticles (58–75%) [28], [29], [54].

We also compared the proportion of different APC populations internalizing CPMV particles following oral, intravenous or intraperitoneal administration. The percentage of APCs containing CPMV particles in the spleen following oral gavage was 10 to 100 fold lower in comparison with i.v. or i.p. routes of inoculation. This phenomenon is likely due to a smaller percentage of the particles transported across the intestinal epithelium. In addition, the lower signal could partly be due to the 20–40% removal of the particle-dye linkage we previously observed in the gastrointestinal tract [26]. At the same time it is possible that APCs present in the intestinal epithelium capture CPMV particles and remain in the PP or migrate to the mesenteric lymph nodes (MLN), thus decreasing the amount of free particles in circulation.

In this study, we showed that CPMV co-localized with M cells and cells within the PP in intestinal epithelium. We had previously demonstrated the ability of CPMV to access the mammalian systemic circulation and enter into a variety of tissues following oral administration, including the spleen [26]. However, the mechanism of translocation across the intestinal epithelia was unknown, and these studies suggest that such translocation occurs via the PP. In general there are three major routes that viruses, bacteria, parasites and macromolecules use to cross the epithelial mucosa: the transcellular route, the paracellular route across cell-to-cell junctions including tight junctions, and via M cells [55]. M cells are specially differentiated epithelial cells in PP, which are located along the antimesenteric side of the small intestine and are most prominent in the terminal ileum. M cells are specialized in capturing and delivering macromolecules, particles, and pathogens into the subepithelial dome region of PP, an area rich in dendritic cells. M cells efficiently transport latex particles, polystyrene microparticles, and viruses including reovirus type 1, HIV type 1 and mouse mammary tumor virus, poliovirus type 1 [55]. Interestingly, both poliovirus and CPMV are structurally-related members of the picornavirus superfamily, and their conserved structural properties may facilitate M-cell interactions.

Once the antigens are transported by the M cells, the first cells that the antigen encounters are most likely DCs present just underneath or within the follicle-associated epithelium of the PP. In the present study we showed that DCs present in the PP SED captured CPMV particles *in vivo*. Oral administration of virus-sized fluorescent polystyrene microparticles to mice results in detection of fluorescent signal in double negative DCs in the SED, and none in the lymphoid or myeloid populations [56]. When DCs are activated after antigen ingestion they may migrate to other zones in the PP or to the MLN. Polystyrene microparticles by themselves do not induce migration, as they remain in the SED for up to 14 days [56]. However, DCs loaded with polystyrene microparticles migrate into T-zones and to B-cell follicles in the PP upon cholera toxin treatment. DCs migration from PP to MLN has also been detected in mice after ingestion of bacteria along with fluorescent particles [57]. We do not know if CPMV particles induce migration of PP DC.

As we have shown in this study, DCs, macrophages and B cells efficiently capture CPMV particles. CPMV chimeras displaying foreign antigenic epitopes have been successfully used as vaccines to prime B-cell responses, but it has not been possible to prime T-cell responses using CPMV as had been reported for several other virus-like particles (PPV [51] and papaya mosaic virus [14]). Thus, in order to prime T-cell responses a more specific understanding of the uptake mechanisms and processing of CPMV-displayed epitopes in comparison to other VLPs is appropriate. Further studies to characterize the CPMV-APC interaction will provide the essential information required for design and development of CPMV based vaccine systems.

## Materials and Methods

### Mice

Adult female C57 BL/6 (H-2b) mice were purchased from the Scripps Research Institute breeding colony and housed in specific pathogen-free conditions. All animal studies were approved by the Scripps Institutional Animal Care and Use Committee (IACUC).

### Cell Culture

MC57 fibroblast cells were grown in RPMI-1640 supplemented with 7–10% heat-inactivated fetal bovine serum (FBS), 2 mM L-glutamine, 100 U/ml penicillin G, and 100 µg/ml streptomycin (all from Gibco-BRL, Rockville, Md.). BalbCl7 fibroblasts and Caco-2 cells were grown in minimum essential media (MEM) with the above supplements. Bone-marrow derived DCs were isolated and cultured following the procedure of Lutz et al. [46].

### Propagation and Purification of CPMV

CPMV resuspended at 25 ng/µl in 0.1 M K-phosphate buffer was used as inoculum for passage to new cowpea plants to produce virus-working stocks. CPMV was purified from the infected leaves by standard methods described by Rae et al. [26]. The final virus pellets were resuspended in PBS (Gibco-BRL) and filter-sterilized through a 0.2 µm membrane (Costar). Virus concentration was measured spectrophotometrically. The CPMV images from wild-type virus particles and the CPMV-vEFα chimera were created using the Visual Molecular Dynamics software [58].

### Chemical Coupling of Fluorescein and AlexaFluor 488 Dyes to CPMV

Fluorescein-5-maleimide dye (Molecular Probes) was coupled to cysteines on the coat protein of the CPMV-vEFα chimera (gift from Dr. J. Johnson) as previously described by Wang et al. [20] with the following modifications. The dye (2.28 mg) was resupended in 1 ml of DMSO and mixed with 5 mg of CPMV in 0.1 M K-phosphate buffer (molar ratio of 100 dyes per asymmetric large-small coat protein unit) in 5 ml final volume and incubated 72 hours at 4°C. To conjugate dyes to lysines on wild-type CPMV, 1 mg Alexa fluor 488 carboxylic acid, succinimidyl ester (Molecular Probes) was resuspended in 0.1 M K-phosphate buffer and mixed with 5 mg of CPMV in a total volume of 1 ml of the same buffer using a molar ratio of 30 dyes per asymmetric large-small coat protein unit. The virus-dye suspension was incubated at room temperature in a rolling shaker for 24–72 hours. After incubation the samples were initially purified by ultracentrifugation (Beckman Coulter, L-90K ultracentrifuge) in a 50.2 Ti rotor at 42,000 rpm (3 hours, 4°C) and resuspended in 1 ml of the same buffer. To eliminate free dye the sample was further purified by a sucrose gradient (30%–10%) ultracentrifugation in an SW-28 rotor at 28,000 rpm (2 hours, 4°C). After collecting the labeled CPMV fractions the virus was concentrated by ultracentrifugation in a 50.2 Ti rotor at 42,000 rpm (3 hours, 4°C). The final pellet was resuspended in PBS (Gibco-BRL) and filtered through a 0.2-µm membrane (Costar) to eliminate particle aggregates. The dye concentration was obtained measuring the absorbance of the sample at 495 nm and using the molar extinction coefficient (∈) of the dye; 86000 for fluorescein and 71000 for AF488 respectively. The number of dyes per virus particle obtained was 71.33/virus for Alexa Fluor 488 and 23/virus for fluorescein, where dyes/particle = Abs_495_×dilution×MW of CPMV/∈×g of CPMV, where CPMV MW = 5.6×10^6^ g/mol. CPMV-AF488 particles used for the combined vimentin and CPMV uptake studies using BalbCl7 mice resulted in 150 dyes/virus particle.

### CPMV Binds to Cell Surface of APCs

The cell lines MC57 and BalbCl7, and primary DCs were cultured in 6 well plates, fixed with paraformaldehyde and incubated with CPMV-AF488 (6.8 µg/ml) overnight at 4°C. After extensive washing with PBS, cells were stained with DAPI and visualized with a Zeiss Axiovert S100 immunofluorescent microscope (20X objective). For FACS analysis, 9 or 10 days differentiated DCs were added to a V-bottom 96 well plate (5×10^5^ cell/well) and incubated with 1 µg of CPMV-AF488 particles (1×10^8^ particles) for 3 hours on ice. Then cells were stained with the antibodies allophycocyanin (APC) anti-CD11c (clone N418) from eBioscience Inc., R-Phycoerythrin (R-PE) anti-CD11b (Mac-1; clone M1/70), R-PE anti-CD86 (B7.2; clone GL1) and R-PE anti-CD80 (B7.1; clone 16-10A1) from BD PharMingen, fixed using formaldehyde 2% in PBS and acquired by FACS. For the binding competition assay 1×10^6^ or 5×10^5^ DCs were stained with the above anti-CD11b and anti-CD11c antibodies and incubated overnight on ice with different amounts of unlabeled CPMV (0 µg, 1 µg, 10 µg, 100 µg, and 1000 µg). After washing and incubating with 1 µg of CPMV-AF488 for 3 hours on ice, the cells were washed, fixed using formaldehyde 2% in PBS, acquired on a FACSort flow cytometer (50,000 events) and analyzed with FlowJo software (Treestar, San Carlos, CA).

### Surface Vimentin Expression

For flow cytometry, the cell lines MC57, BalbCl7, Caco-2, and RAW macrophages were cultured in flasks and collected into V-bottom 96 well plates (1−5×10^5^ cells/well). Splenocytes and Peyer's patch cells were collected from mice as previously described. Cells were stained with vimentin antibody V4630 followed by staining with Alexafluor 647 conjugated to donkey anti-goat antibody. They were fixed with 2% EM grade formaldehyde for 15 minutes and then acquired on a FACSort flow cytometer (10,000 events) and analyzed with FlowJo software (Treestar, San Carlos, CA).

For confocal microscopy, Caco-2 cells were seeded into glass-bottom culture dishes (Mat-tek) at 50,000–100,000 cells in 3 ml media per dish and grown overnight. The following day, cells were fixed for 10 minutes at room temperature in 3% EM grade formaldehyde and 0.3% gluteraldehyde in PBS. Cells were washed and blocked for one hour in PBS with 5% normal goat serum (NGS). Cells were then incubated with vimentin antibody V9 (1∶100) for 1.5 hours in 1% NGS PBS. Cells were washed and secondary antibody, chicken anti-mouse conjugated to Alexafluor-647 (1∶200) was added in 1% NGS PBS for one hour. Cells were washed and incubated one hour with wheat germ agglutinin conjugated to Alexafluor-555 (1∶500) in 1% NGS PBS. During the final 10 minutes, DAPI (1∶9500) was added in PBS. Cells were washed, and the cells were mounted in Immunofluore Mounting Medium. Cells were visualized at 60x with a Bio-Rad Radiance 2100 Rainbow laser scanning confocal microscope. All incubations take place at room temperature on a slow rock. Following each incubation, cells were washed four times in PBS in order to remove antibody that is bound nonspecifically.

### Cellular Uptake of CPMV In Vitro

MC57 and BalbCl7 cells (200,000 and 80,000 cells/well respectively) were cultured on Lab-Tek II chamber polylysine slides (Nalge Nunc Naperville, IL) and CPMV-FITC was added to the media. DCs were cultured in 24 well plates with CPMV-AF488. Cultures were incubated overnight at 37°C and 5% CO_2_. Cells were washed 3 times with PBS, fixed with 2% formaldehyde, stained with DAPI and visualized with a Zeiss Axiovert S100 immunofluorescence microscope. DCs were stained with the specific antibodies markers as indicated above. Cells were fixed using 2% formaldehyde in PBS, acquired on a FACSort flow cytometer (30,000 events per sample) and analyzed with FlowJo software (Treestar, San Carlos, CA).

For correlation of CPMV uptake and presence of surface vimentin, BalbCl7 cells were cultured and incubated with CPMV-AF488 for 3 and 24 hours at 37°C and 5% CO_2_. Cells were washed and fixed in 2% formaldehyde and incubated with antibodies specific for vimentin. After washing, the cells were stained with a secondary antibody, washed, and acquired on a BD Digital LSR II and analyzed with FlowJo software.

### Cellular Uptake of CPMV In Vivo

Groups of 3 mice each were inoculated i.v. or i.p. with 100 µg or by oral gavage with 1 mg of CPMV-AF488. Negative control mice were inoculated with PBS. Four hours after i.v. or i.p. and 20 hours after oral gavage mice were sacrificed and the spleens harvested. The spleens were injected with 1 ml solution of 1 mg/ml of Collagenase-D (ROCHEeim) in RPMI medium, then cut in small pieces and incubated at 37°C for 15 min. To disrupt T-cell-DC complexes 4 µl of 0.5 M EDTA were added to the cell suspension and incubated at 37°C/5 min. After collagenase-D treatment a single cell suspension of splenocytes were prepared according to standard procedures. The cells (2×10^6^) were washed once with FACS buffer (1% FBS and 0.1% Na-azide in PBS) and nonspecific binding was blocked for 10 minutes on ice using rat anti-mouse CD16/CD32 antibody in FACS buffer (clone 2.4 G2, BD PharMingen). The cells were stained with the following rat anti-mouse monoclonal antibodies: APC anti-CD11c (clone N418) and PE anti-NK 1.1 (clone PK136) from eBioscience; R-R-PE anti-CD8α (clone 53–6.7), R-PE anti-CD11b (clone M1/70), R-PE anti-CD45R/B220 (clone RA3-6B2) from BD PharMingen and fixed in 2% formaldehyde in PBS. Cells (100,000 events per sample) were acquired on a FACSCalibur flow cytometer (Becton Dickson, San Jose, CA) and analyzed with FlowJo software (Treestar, San Carlos, CA). Spleen cells from i.p. inoculated mice were also visualized by fluorescence microscopy as described above.

### Ileal Loop Inoculation with CPMV-AF488 and PP Analysis

C57 BL/6 mice were anesthetized with an i.p. injection of 500 µl Avertin (2,2,2 tribromoethanol) solution at 20 mg/ml. Intestinal loops of approximately four cm containing a single PP were constricted and inoculated with 200 µg CPMV-AF488 and 20 µg UEA Rhodamine (Vector Laboratories) M cell binding lectin in a PBS solution of 450 µl total volume. Mice were incubated under anesthesia and heat for one hour. Following incubation, the loops were harvested and gently flushed with PBS. The PP were then either isolated for flow cytometry analysis or mounted in OCT medium for cryosectioning. For flow cytometry analysis PP were inoculated only with CPMV-AF488 treated with collagenase D to prepare a single cell suspension and stained the same way as described for the spleens, but using the antibody markers, APC-CD11c, PE-CD11b and Peridinin Chlorophyll-a protein (PerCP)-CD8a (clone 53–6.7) (BD Pharmingen). Negative controls were inoculated with PBS. For cryosectioning the loops were inoculated with both CPMV and UEA and six micron cryosections were made in a Leica cryomicrotome. The sections were fixed in acetone, subjected to DAPI nuclear stain and analyzed under a fluorescence microscope (Nikon Eclipse TS100) using the 20X objective.

## Supporting Information

Figure S1 Space-filling model of the CPMV structure. Created using Visual Molecular Dynamics software (VMD) [57] showing the S protein in green and the L protein in light grey. (A) Naturally occurring exposed lysines are highlighted in red. (B) Cysteine residues of a genetically modified CPMV (vEFα) particle are highlighted in blue.
(0.79 MB TIF)

Figure S2 Correlation of surface vimentin expression and ability to take up CPMV. Flow cytometry profiles of BalbCl7 cells, both unstained, non-incubated (A), and incubated with CPMV-AF488 (B–D). Cells in C and D were stained with primary vimentin antibody and secondary, while B represents a secondary only control. B and C were incubated with CPMV for 3 hrs, and D for 24 hrs.
(1.44 MB TIF)

Figure S3 CPMV uptake by different subpopulation of APCs in vivo. Flow cytometry profiles of splenocytes from noninoculated mice and mice inoculated intraperitonealy with CPMV-AF488. The figure shows the uptake profile of different subpopulations of splenocytes.
(2.86 MB TIF)
