# Supplementary figures and images for: Interaction of Cowpea Mosaic Virus (CPMV) Nanoparticles with Antigen Presenting Cells In Vitro and In Vivo
Source: PLoS One. 2009 Nov 23;4(11):e7981. doi: 10.1371/journal.pone.0007981 (PMC2776531; doi:10.1371/journal.pone.0007981)

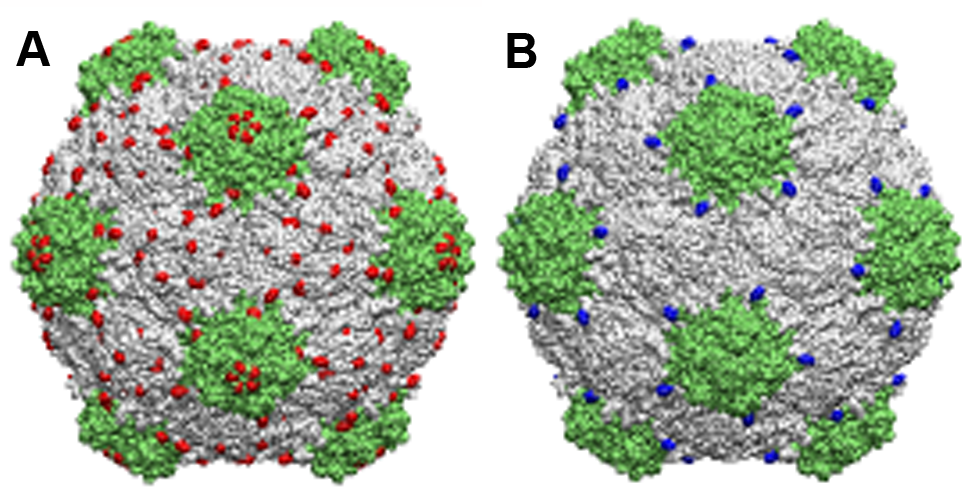

Supplement: Figure S1 — Space-filling model of the CPMV structure. Created using Visual Molecular Dynamics software (VMD) [57] showing the S protein in green and the L protein in light grey. (A) Naturally occurring exposed lysines are highlighted in red. (B) Cysteine residues of a genetically modified CPMV (vEFα) particle are highlighted in blue. (0.79 MB TIF) [file pone.0007981.s001.tif]

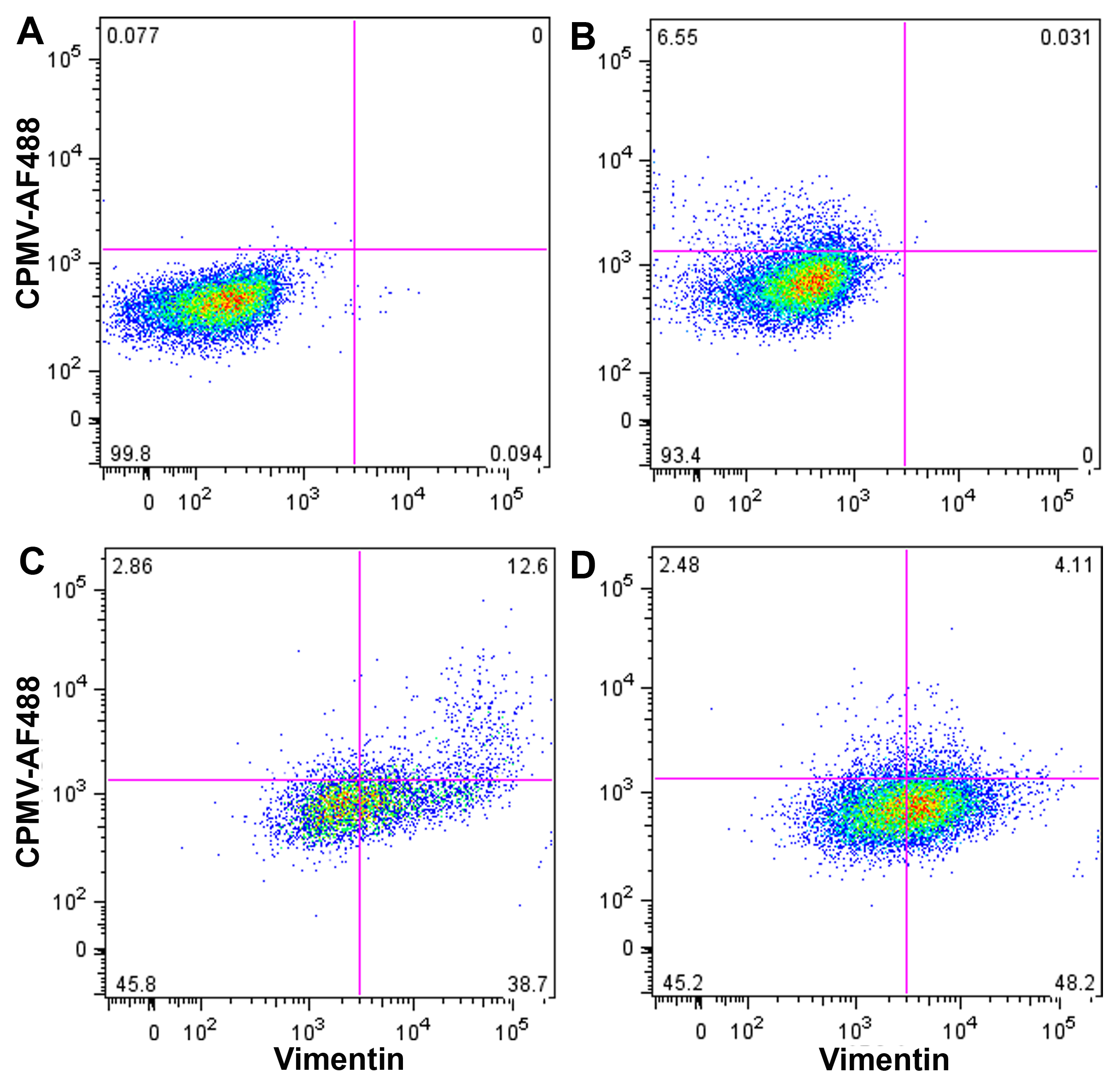

Supplement: Figure S2 — Correlation of surface vimentin expression and ability to take up CPMV. Flow cytometry profiles of BalbCl7 cells, both unstained, non-incubated (A), and incubated with CPMV-AF488 (B–D). Cells in C and D were stained with primary vimentin antibody and secondary, while B represents a secondary only control. B and C were incubated with CPMV for 3 hrs, and D for 24 hrs. (1.44 MB TIF) [file pone.0007981.s002.tif]

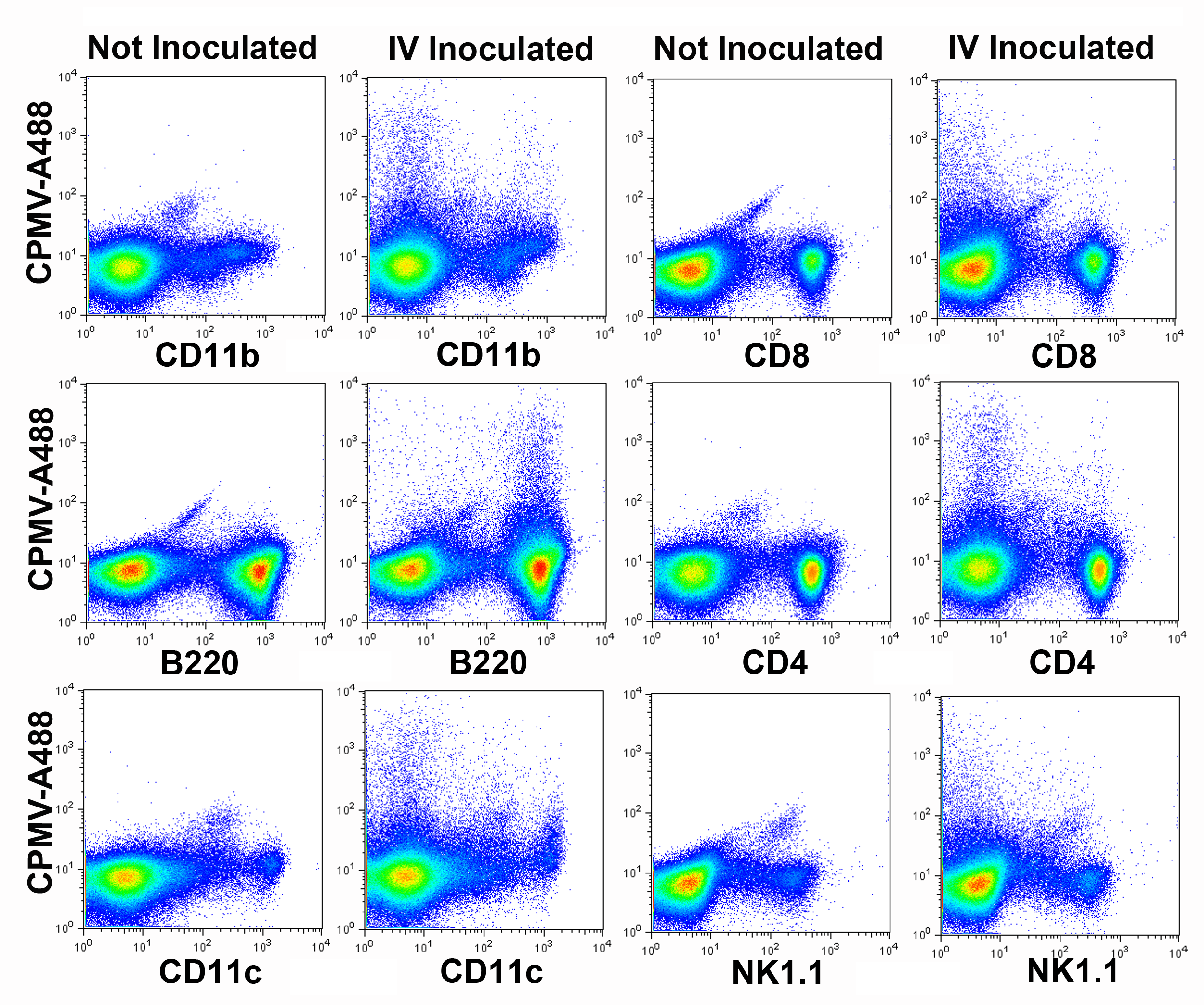

Supplement: Figure S3 — CPMV uptake by different subpopulation of APCs in vivo. Flow cytometry profiles of splenocytes from noninoculated mice and mice inoculated intraperitonealy with CPMV-AF488. The figure shows the uptake profile of different subpopulations of splenocytes. (2.86 MB TIF) [file pone.0007981.s003.tif]
